# Prior elicitation of the efficacy and tolerability of Methotrexate and Mycophenolate Mofetil in Juvenile Localised Scleroderma

**DOI:** 10.12688/amrcopenres.13008.1

**Published:** 2021-09-09

**Authors:** Yasin Desai, Thomas Jaki, Michael W Beresford, Thomas Burnett, Despina Eleftheriou, Heidi Jacobe, Valentina Leone, Suzanne Li, Pavel Mozgunov, Athimalaipet V Ramanan, Kathryn S Torok, Marina E Anderson, Jordi Anton, Tadej Avcin, Jessie Felton, Ivan Foeldvari, Bisola Laguda, Flora McErlane, Lindsay Shaw, Francesco Zulian, Clare E Pain

**Affiliations:** 1 MPS Research Unit, Department of Mathematics and Statistics, Lancaster University, Lancaster, LA1 4YF, UK; 2 MRC Biostatistics Unit, University of Cambridge, Cambridge, CB2 0SR, UK; 3 Department of Paediatric Rheumatology, Alder Hey Children’s NHS Foundation Trust, Liverpool, L12 2AP, UK; 4 Institute of Life Course and Medical Sciences, University of Liverpool, Liverpool, L69 3BX, UK; 5 University College London Great Ormond Street Institute of Child Health, London, WC1N 1EH, UK; 6 Department of Paediatric Rheumatology, Great Ormond St Hospital NHS Foundation Trust, London, WC1N 3JH, UK; 7 UT Southwestern Medical Center, Dallas, Texas, TX 75390, USA; 8 Paediatric Rheumatology Department, Leeds Children Hospital (Leeds Teaching Hospitals) and University of Leeds, Leeds, LS1 3EX, UK; 9 Department of Pediatrics, Joseph M. Sanzari Children’s Hospital, Hackensack University Medical Center & Hackensack Meridian School of Medicine, Hackensack, New Jersey, NJ 07601, USA; 10 University Hospitals Bristol NHS Foundation Trust & Translational Health Sciences, Bristol, BS1 3NU, UK; 11 Division of Rheumatology, Department of Pediatrics, Children's Hospital of Pittsburgh, University of Pittsburgh, Pittsburgh, PA 15260, USA; 12 Department of Rheumatology, Liverpool University Hospitals NHS Foundation Trust, Liverpool, L9 7AL, UK; 13 Lancaster Medical School, Lancaster University, Lancaster, LA1 4YF, UK; 14 Pediatric Rheumatology, Hospital Sant Joan de Déu, University of Barcelona, Barcelona, Barcelona, 08007, UK; 15 Department of Allergology, Rheumatology and Clinical Immunology, University Children's Hospital, University Medical Centre, Ljubljana, 1000 Ljubljana, Slovenia; 16 Department of Dermatology, Brighton and Sussex University Hospitals & Royal Alexandra Children’s Hospital, Brighton, BN2 1DH, UK; 17 Hamburg Centre for Pediatric and Adolescence Rheumatology, Hamburg, 22081 Hamburg, Germany; 18 Department of Paediatric Dermatology, Chelsea and Westminster Hospital, London, SW10 9NH, UK; 19 Department of Paediatric Rheumatology, Great North Children's Hospital, Newcastle, NE1 4LP, UK; 20 Department of Woman's and Child's Health, University of Padova, Padua, 35122 Padua, Italy

**Keywords:** juvenile localised scleroderma, methotrexate, mycophenolate mofetil, Bayesian approach, prior elicitation

## Abstract

**Background:**

Evidence is lacking for safe and effective treatments for juvenile localised scleroderma (JLS). Methotrexate (MTX) is commonly used first line and mycophenolate mofetil (MMF) second line, despite a limited evidence base. A head to head trial of these two medications would provide data on relative efficacy and tolerability. However, a frequentist approach is difficult to deliver in JLS, because of the numbers needed to sufficiently power a trial. A Bayesian approach could be considered.

**Methods:**

An international consensus meeting was convened including an elicitation exercise where opinion was sought on the relative efficacy and tolerability of MTX compared to MMF to produce prior distributions for a future Bayesian trial. Secondary aims were to achieve consensus agreement on critical aspects of a future trial.

**Results:**

An international group of 12 clinical experts participated. Opinion suggested superior efficacy and tolerability of MMF compared to MTX; where most likely value of efficacy of MMF was 0.70 (95% confidence interval (CI) 0.34-0.90) and of MTX was 0.68 (95% CI 0.41-0.8). The most likely value of tolerability of MMF was 0.77 (95% CI 0.3-0.94) and of MTX was 0.62 (95% CI 0.32-0.84). The wider CI for MMF highlights that experts were less sure about relative efficacy and tolerability of MMF compared to MTX. Despite using a Bayesian approach, power calculations still produced a total sample size of 240 participants, reflecting the uncertainty amongst experts about the performance of MMF.

**Conclusions:**

Key factors have been defined regarding the design of a future Bayesian approach clinical trial including elicitation of prior opinion of the efficacy and tolerability of MTX and MMF in JLS. Combining further efficacy data on MTX and MMF with prior opinion could potentially reduce the pre-trial uncertainty so that, when combined with smaller trial sample sizes a compelling evidence base is available.

## List of abbreviations

CARRA, Childhood Arthritis & Rheumatology Research Alliance;

CI, confidence intervals;

CS, corticosteroids;

DMARD, Disease Modifying Anti-Rheumatic Drug;

IQR, interquartile range;

JLS, juvenile localised scleroderma;

LoSCAT, localized scleroderma cutaneous assessment tool;

LoSQI, Localised Scleroderma Quality of Life Instrument;

LS, localised scleroderma;

MAC, Morphea in Adults and Children cohort;

mLoSSI; modified localised scleroderma skin severity index;

MMF, mycophenolate mofetil;

MTX, methotrexate;

NRCOS, National Registry of Childhood Onset Scleroderma;

p _MTX_, probability of successful treatment with methotrexate;

p _MMF_, probability of successful treatment with mycophenolate mofetil;

RCT, randomised controlled trial;

RPCT, randomised placebo controlled trial;

RIO, rational impartial observer;

t _MTX, _probability of tolerating methotrexate;

t _MMF, _probability of tolerating mycophenolate mofetil;

UK, United Kingdom

## Background

Juvenile localised scleroderma (JLS) is characterised by chronic inflammation of the skin and adjacent tissue leading to fibrosis ^1^. It is associated with significant complications that impact quality of life ^2^. The incidence rate of JLS in the UK is 3.4 per one million children per year and has a prevalence from 3.2 to 3.6 per 10 000 children ^3, 4^.

Treatment of JLS remains challenging. To date there has only been one randomised trial in JLS which was a randomised placebo-controlled trial (RPCT) of MTX ^5^. At one year, a disease relapse rate of 32.6% was shown in the MTX group compared to 70.8% of the placebo group (p<0.005). This highlighted that MTX therapy is superior to placebo but may not be effective in controlling disease in one third of patients. In addition, significant side effects are associated with MTX ^5– 7^ including gastro-intestinal symptoms, such as nausea, vomiting (20% in the MTX arm of the RPCT) and anticipatory vomiting (20% in a North American prospective JLS cohort) ^8^. A UK wide study showed that children discontinuing MTX for JLS, reported medication side-effects as the primary reason ^9^.

An emerging alternative to MTX is MMF ^10, 11^. Clinical practice internationally confirms the common use of MMF in those that have failed MTX, despite a limited evidence base ^12, 13^. MMF reduces B and T lymphocyte proliferation ^14^, attenuates fibrosis *in vitro* ^15^, and *in vivo* by mitigating the inflammatory gene expression and skin score in scleroderma ^16^. Therefore, it may have an effect on both the inflammatory and fibrotic aspects of this disease. A growing body of data from high-quality clinical trials in adults suggests that MMF is a well-tolerated alternative for the induction of remission of different autoimmune diseases ^17– 20^. The use of MMF in JLS may significantly reduce the burden of disease and drug toxicity.

Several evidence-based guidelines on the management of JLS have been published ^21– 27^. All include MTX as first line treatment together with corticosteroids (CS) and most suggest second line treatment with MMF. Multiple regimes for CS have been described, including both oral and intravenous ^5, 8, 13, 28– 31^. Consensus treatment plans formulated by the Childhood Arthritis and Rheumatology Research Alliance (CARRA) define three CS arms and the use of MTX first line with MMF as second line or add on therapy ^26^.

Given the confirmed efficacy of MTX but with poor associated tolerability, and limited evidence base for MMF, a head to head study of MTX against MMF is warranted to allow direct comparison of the efficacy and tolerability of the two treatments. This would provide data to inform the usefulness (or otherwise) of MMF but also may demonstrate that MMF is potentially preferable as first line treatment. Unfortunately, a frequentist trial that compares these two agents would require large numbers of patients which is not feasible, even with international collaboration, in a rare disease like JLS. A frequentist trial design would require a total sample size of 320 patients, assuming a non-inferiority margin of 15%, and (for example) a one-sided type-I error of 5% and 90% power, if both treatments have a 70% response rate. With the number of new cases in the UK per year being estimated at 43, it would take at least 15 years to complete the study, presuming a 50% consent rate in all centres ^3^. An alternative to the conventional frequentist statistical methodology is the Bayesian framework for the design and analysis of a clinical trial ^32^. Bayesian methodology utilises data from a randomised controlled trial (RCT) alongside prior opinion from an expert group of the efficacy and tolerability of each drug. This prior opinion can be informed from multiple sources including clinical experience of the experts in using the two medicines, and evidence from published and unpublished datasets. As MMF is commonly used second line, a clinical trial using a Bayesian approach is appealing because experts will have clinical experience of using both drugs and observational data sources exist to inform prior opinion.

By utilising the prior information in conjunction with the trial data, the required sample size can be reduced provided that the prior information agrees with the trial data whilst still achieving desirable operating characteristics. If, however, the prior information is in conflict with information from the subsequent trial, conclusions may be less precise. Therefore, this methodology cannot, and should not, be used *ad hoc* to lower required sample size.

In this work we describe the underlying trials methodology and report the results of an international consensus meeting to establish the pre-trial evidence base of the two treatments. Here, we utilised expert opinion to elicit prior opinion, an approach that has been previously used in paediatric rheumatology clinical trials such as the MYPAN trial ^33, 34^. On the basis of these results we developed the design of a potential future trial comparing MMF to MTX.

## Methods

### Synthesis of data from published and unpublished sources to define primary outcome and inform prior elicitation

A study group of experts (CP, DE, SL, HJ, KT, VL, YD, TJ) met frequently via teleconference to discuss relevant data from pre-existing databases to define what constitutes successful treatment and tolerance. A scoping exercise for available tools for measuring tolerability was performed. A literature review of efficacy and tolerability of MTX and MMF was undertaken by systematic review researchers at Keele University, UK (Jo Jordan, Nadia Corp).

### Establishing a group of experts to determine consensus prior opinion

An electronic survey was sent to potentially eligible clinician’s through a number of national/international societies and professional organisations. Direct invitations were sent to 9 individuals who had published sentinel research on JLS. All participants of the prior elicitation meeting were clinicians, chosen on the basis of pre-defined definitions of a clinical expert, ensuring representation from UK and non-UK sites within the constraints of the funding of the study. Definition of clinical expert, identification of eligible clinicians, response to the survey and methodology for selection of experts can be found in extended data in the Data Availability section.

### Establishing consensus on key elements of a future RCT and prior opinion of efficacy & tolerability of MTX and MMF

**Figure 1.**
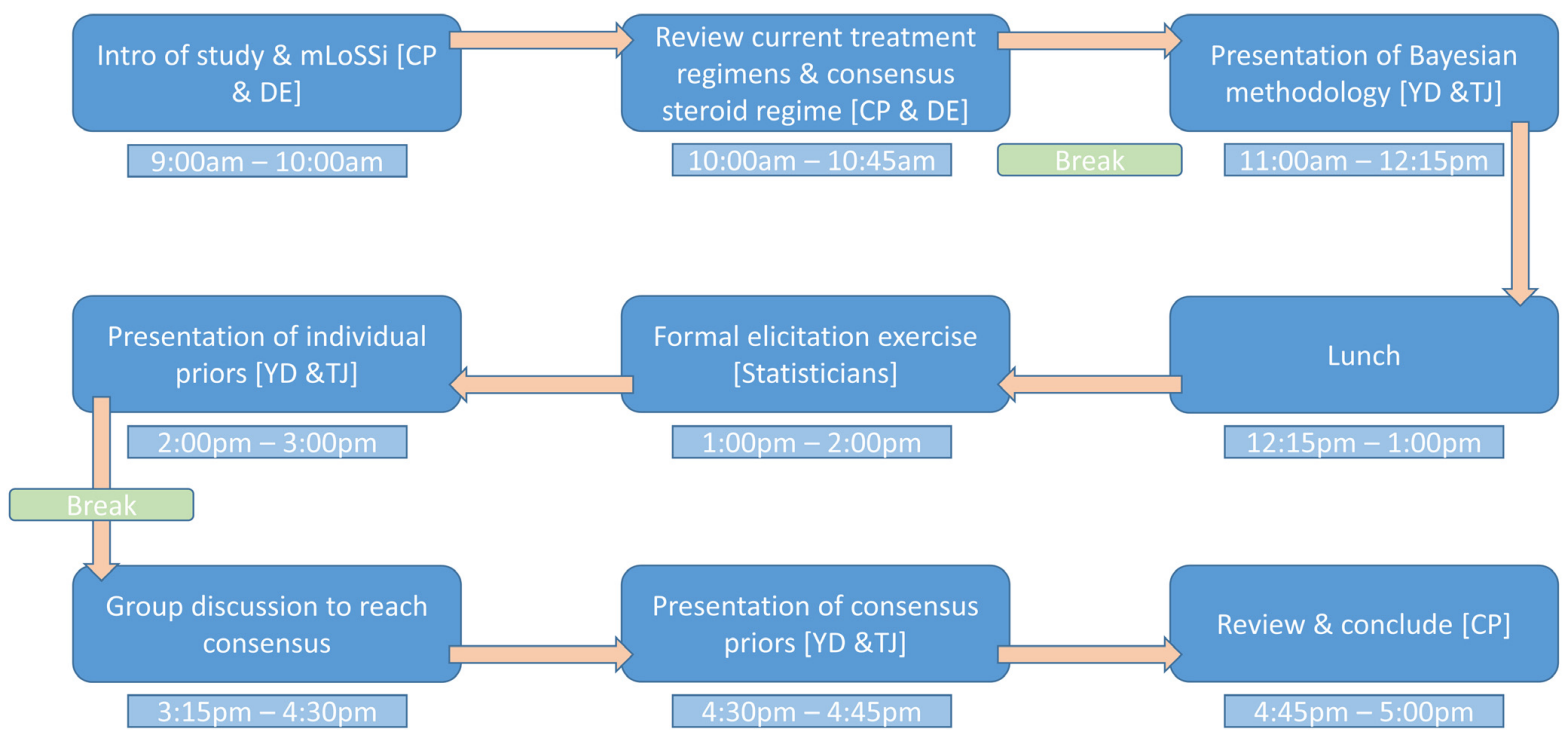
Outline of events for the prior elicitation meeting.

Figure 1 outlines the agenda of the prior elicitation day. Participants were sent preparatory material before the meeting summarising key elements of a future proposed trial in JLS and current available evidence for efficacy and tolerability of the two medicines. Before any elicitations could take place, a recap of current evidence of efficacy and tolerability of MTX and MMF was presented together with evidence of CS regimes. Consensus agreement was sought on certain key elements of a clinical trial including primary outcome definition and CS regimes.

The consensus meeting was conducted under the premise that the future trial will be a multi-centre randomised controlled open label study of MMF and CS compared to MTX and CS in children and young adults with active JLS using a Bayesian design. It was stipulated that randomisation would be 1:1 between MTX 15mg/m2 by oral or subcutaneous route once weekly (max 25mg weekly) together with a CS induction regime (to be confirmed) and MMF 600mg/m2 orally twice daily (maximum 2g daily) and CS induction regime (to be confirmed). Predefined inclusion criteria were set before the elicitation exercise (extended data in the Data Availability section).

An introduction to Bayesian methodology followed by an elicitation exercise to formulate prior distributions occurred. Specifically, we were interested in: p _MTX _(probability of successful treatment with MTX), p _MMF _(probability of successful treatment with MMF), t _MTX _(probability of tolerating MTX) and t _MMF _(probability of tolerating MMF).

Experts underwent training in understanding statistical terminology and methods, including probability density functions and Bayesian methodology.

The questions used to elicit and characterise expert’s opinion were based on those used in the MYPAN trial ^32, 33^. These were adjusted to suit this scenario, following the definitions of successful treatment and tolerance defined above (questionnaire provided in extended data in the Data Availability section).

Each expert was asked to provide initial responses. In a one-to-one meeting with a statistician the R Shiny application was used to produce graphical results of the prior distributions using these initial responses to see if the characterisation was according to their expectation ^35, 36^. Alterations were made as required until the resulting distributions adequately reflected the individual’s opinion.

Once all the individual prior distributions were obtained, they were presented to all experts collectively. Behavioural aggregation method was used to arrive at consensus through a discussion-based method. Any distributions that significantly differed to the rest were highlighted to further probe the group to come to a decision.

A rational impartial observer (RIO) was used from the Sheffield Elicitation Framework, to eliminate the idea of the consensus distribution becoming one expert’s opinion ^37^.

### Statistical approach for establishing Bayesian prior distributions

The R Shiny package was used to create the application, modifying the application used in the MYPAN study ^32, 33, 35, 36^. 

Prior distributions were elicited on the efficacy and tolerability of MTX, which was viewed as the control treatment. The endpoints for efficacy/tolerability were modelled as binary (i.e. success for treatment – Yes/No and tolerated treatment – Yes/No) and the log odds ratio was used to compare the two treatments for efficacy, θ _efficacy_, and tolerability, θ _tol_. We define θ _efficacy _and θ _tol_ as

${\text{θ}}_{\text{efficacy}}\;=\text{log}\;\left(\frac{{\text{p}}_{\text{MMF}}\;(1-{\text{p}}_{\text{MTX}})}{{\text{p}}_{\text{MTX}}\;(1-{\text{p}}_{\text{MMF}})}\right)\;{\text{θ}}_{\text{tol}}\;=\text{log}\;\left(\frac{{\text{t}}_{\text{MMF}}\;(1-{\text{t}}_{\text{MTX}})}{{\text{t}}_{\text{MTX}}\;(1-{\text{t}}_{\text{MMF}})}\right)$

such that positive values indicate MMF is superior to MTX and negative values correspond to better outcomes on MTX over MMF.

The parameters p _MTX_, t _MTX_, θ _efficacy_, θ _tol_ were elicited directly whilst the prior distributions of p _MMF_ and t _MMF,_ were calculated analytically through a numerical integration algorithm. This allowed the pair of parameters (p _MTX,_ θ _efficacy_) to be treated as independent of each other ^32, 33^.

p _MTX_ and t _MTX_ were modelled as beta distributions while Gaussian prior distributions were used to model θ _efficacy _and θ _tol_.

### Ethical approval

Ethical approval was not required as participants volunteered to take part as experts and no patients were involved.

## Results

### Establishment of primary outcome measure and consensus agreement on key elements of trial design

The Localised Scleroderma Cutaneous Assessment Tool (LoSCAT) is a validated measure in JLS which combines activity (mLoSSI) and damage (LoSDI) components ^38, 39^. It is an easy to use skin score which can be completed by clinicians in the clinic room without specialist equipment. Reliability has been tested in several cohorts including adults and as an outcome measure in clinical trials ^8, 40– 44^. The mLoSSI activity component was chosen as the measure of efficacy for the consensus meeting. However, a study comparing efficacy alone may not address patient need. Patients and families in the UK expressed the need for more tolerable treatments, specifically related to intolerance from MTX (unpublished data from Scleroderma & Raynaud’s UK family day). We therefore agreed a composite primary outcome measure of efficacy and tolerability could be advantageous, hypothesising that whilst efficacy may be similar, tolerability may be better with MMF.

Choice of this composite primary outcome was made after review of pre-existing databases. These databases included: the National Registry of Childhood Onset Scleroderma (NRCOS) ^8, 41, 45, 46^, the Morphea in Adults and Children (MAC) cohort ^47– 49^, and JLS pilot Consensus Treatment Plan studies ^50^, which combined has longitudinal standardly collected prospective outcome data on over 900 localised scleroderma (LS) subjects (497 JLS, rest adults with LS) ^51^. Average baseline mLoSSI (in all subtypes and specifically in head involvement), expected change in mLoSSI with treatment, minimal mLoSSI for entry into an RCT, time frame for primary outcome measure change and side-effects of MTX and MMF within these cohorts were reviewed.

Efficacy was agreed to be measured by the mLoSSI score, where inactive disease was defined as mLoSSI score of 0 and active disease as mLoSSI of 1 or greater. No ideal tool could be identified to measure tolerability of MTX and MMF. Therefore, a pragmatic definition of intolerance was agreed as the *‘need to permanently stop or reduce dose of a drug because of adverse events (patient reported or laboratory abnormalities) or the need to add in additional medications to counteract side-effects (e.g. anti-emetic)’*. In both cases of efficacy and tolerability, a binary measure occurs with a positive result represented by a 1 and a negative result by 0.

In June 2019, twelve international clinicians with expertise in JLS including 3 dermatologists attended the face-to-face consensus meeting (extended data Appendix S1). Several key statements required consensus agreement prior to the elicitation process. These are summarised in Table 1.

**Table 1.** Key statements and consensus agreements.

Statement	Consensus Agreement
Primary endpoint * would be measured at 12 months	12/12
Inactive disease is defined as mLoSSI score of 0	12/12
Because of the paucity of evidence in JLS, the route of administration of MTX (oral versus subcutaneous) would not influence the experts prior opinion on efficacy and tolerability of MTX	12/12
Inclusion criteria for the proposed clinical trial should include all subtypes of JLS where a DMARD is deemed necessary by the clinician, specifically isolated linear head lesions can also be included	12/12
Exclusion criteria would include participants where the main indication for starting a DMARD was due to extra-cutaneous manifestations such as uveitis or arthritis as the primary efficacy outcome (mLoSSI) only measures skin involvement	12/12

*Primary endpoint binary composite measure of efficacy (as measured by a mLoSSI score of 0) and tolerability (as measured by the absence of the need to permanently stop or reduce dose of a drug because of adverse events (patient reported or laboratory abnormalities) or the need to add in additional medications to counteract side-effects (e.g. anti-emetic). Abbreviations: DMARD, Disease Modifying Anti-Rheumatic Drug; JLS, juvenile localised scleroderma; mLoSSI; modified localised scleroderma skin severity index; MMF, mycophenolate mofetil; MTX, methotrexate

Three CS regimens (A, B, C) were outlined based on a literature review of current evidence. A further regimen (D) was agreed on at the meeting which represented a regimen currently used in practice by several of the experts. After an initial discussion of the merits and demerits of the different regimens, consensus was sought through a process of elimination and voting about the different options. The four regimens that were discussed are given in Table 2 as well as the number of votes to help arrive at consensus. All experts agreed that regimen B was the most acceptable within a clinical trial setting.

**Table 2.** Corticosteroid regimens considered during consensus process.

	A	B	C	D
Route	IV and Oral	Oral	Oral	Oral
Regime	30mg/kg/day IV methylprednisolone for 3 days followed by: Oral prednisolone 1.5mg/kg/ day for 2 weeks (maximum 45mg) then 1mg/kg for 2 weeks (maximum 30mg) then 0.5mg/kg for 2 weeks (maximum 15mg) then 0.25mg/kg/day for 2 weeks (maximum 7.5mg) then stop	Oral prednisolone 1.5mg/kg/day for 2 weeks (maximum 45mg) then 1mg/kg for 2 weeks (maximum 30mg) then 0.5mg/kg for 2 weeks (maximum 15mg) then 0.25mg/kg/day for 2 weeks (maximum 7.5mg) then stop	Oral prednisolone 2mg/kg/ day for 2 weeks (maximum 80mg) then 1.5mg/kg/day for 2 weeks (maximum 45mg) then 1mg/kg for 2 weeks (maximum 30mg) then 0.5mg/kg for 2 weeks (maximum 15mg) then 0.25mg/kg/day for 2 weeks (maximum 7.5mg) then stop	Oral prednisolone 1mg/kg 2 months then reduce to 0.75mg/kg for one week then to 0.5mg/kg for one week then to 0.25mg/kg for one week then stop
Total duration of CS use	3 days IV then 8 weeks oral	8 weeks	10 weeks	11 weeks
Number of experts that would accept this regimen in a clinical trial setting (n=12)	9	12	10	7
First choice of CS regimen chosen by experts (n=12)	2	9	1	0

Abbreviations: CS, corticosteroid; IV, intravenous; kg, kilogram; mg, milligram

### Individual prior distributions

**Figure 2.**
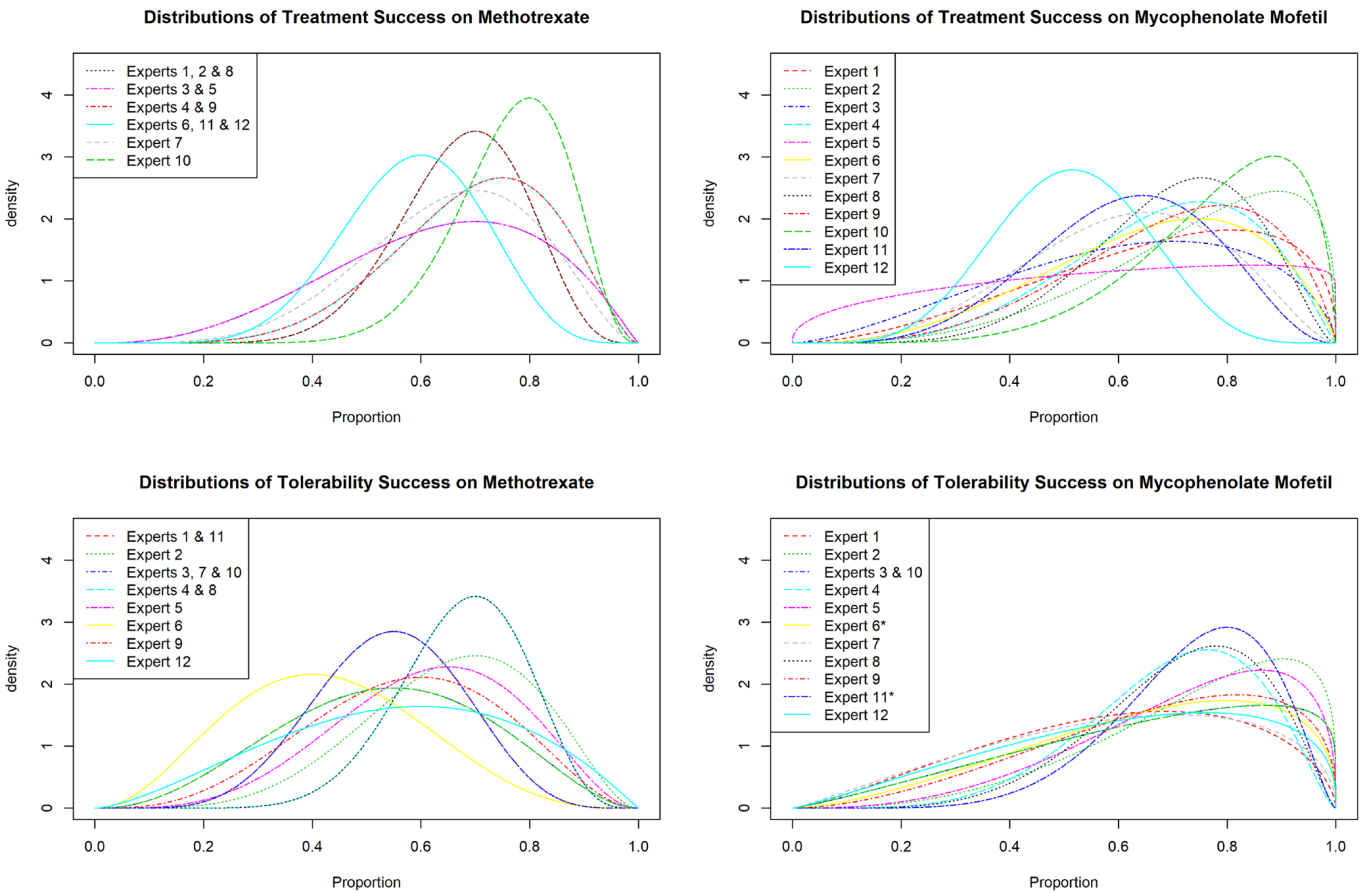
Prior distributions for each expert for MTX (left) and MMF (right) for efficacy (top) and tolerability (bottom).

Figure 2 summarises four plots showing the individual prior distributions about the efficacy and tolerability of MTX and MMF. All opinions on MMF were derived from the log odds ratio to ensure statistical independence except for two results. In both of these cases, the clinician’s opinion on the tolerability of MMF could not be modelled adequately through questions 3 and 4. This was due to the numerical integration not finding a stable solution due to the experts being either themselves or altogether more uncertain in their opinion on the tolerability of MMF. In these cases, the redundant questions provided the information required. This is shown on Figure 2 with stars where applicable.

From Figure 2, the following conclusions were made: experts were more certain about their opinions on MTX rather than MMF, with multiple experts sharing the same prior distributions; experts seem to believe that MMF is superior in terms of tolerability, however they are uncertain by how much it may outperform MTX.

### Consensus prior distributions

**Figure 3.**
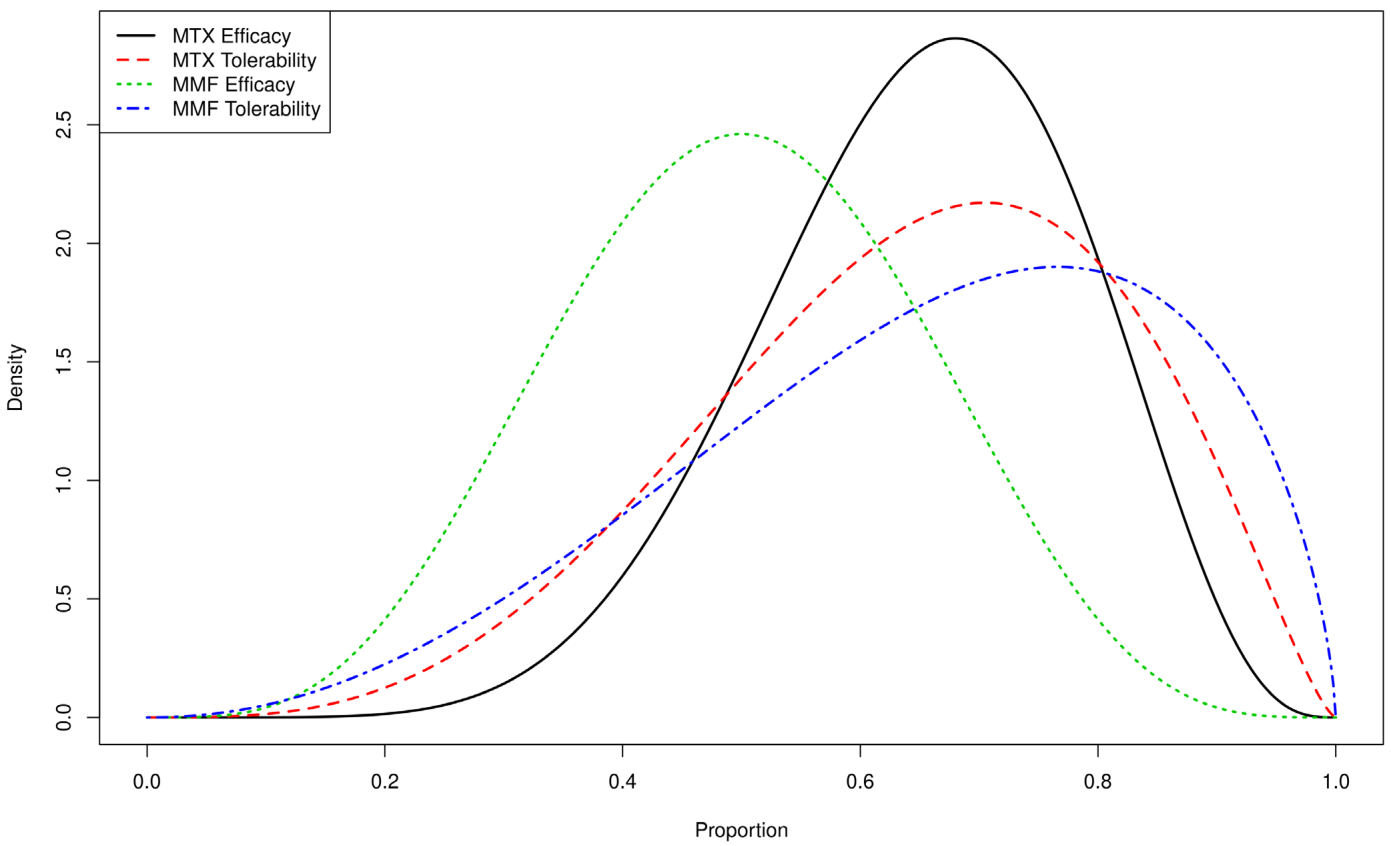
Consensus prior distribution for tolerability and efficacy on MTX and MMF.

Figure 3 provides the consensus prior distributions, whilst Table 3 presents a summary. Based on the consensus answers of the experts, the most likely value of the efficacy of MTX was 68% and most likely value of efficacy of MMF was 70%. The most likely value of tolerability of MTX was 62% and tolerability of MMF 77%.

**Table 3.** Summary of consensus prior distributions.

Quantity of interest	Most likely value	50% CI	95% CI
p _MTX_	0.680	(0.560, 0.748)	(0.416, 0.854)
p _MMF_	0.705	(0.527, 0.777)	(0.339, 0.899)
t _MTX_	0.620	(0.480, 0.711)	(0.316, 0.842)
t _MMF_	0.766	(0.522, 0.813)	(0.300, 0.936)

CI, confidence intervals; p _MTX_ efficacy of methotrexate; p _MMF _efficacy of mycophenolate mofetil; t _MTX_, tolerability of methotrexate; t _MMF_ tolerability of mycophenolate mofetil

**Figure 4.**
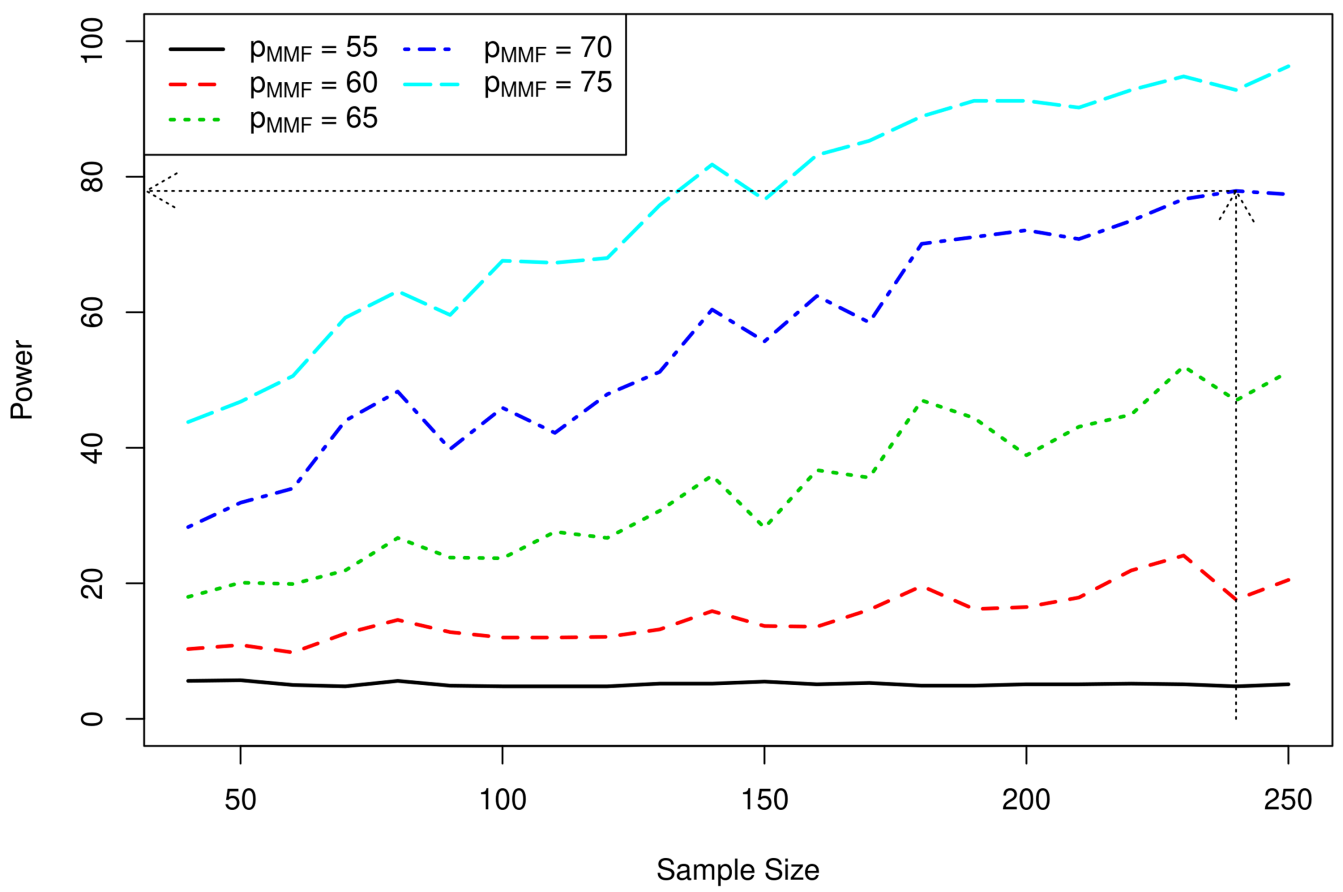
Results of simulations showing potential sample sizes. Legend: pMMF efficacy of mycophenolate mofetil where e.g. pMMF = 55 corresponds to efficacy of 55% (response rate), pMMF = 75 corresponds to efficacy of 75%. The black dotted line shows that a sample size of 240 would yield power of approximately 80%.

We used simulations to explore the properties of different scenarios in order to assess their viability in a future trial. In the 1000-fold simulations, we fixed p _MTX _= 0.7 and assumed equal randomisation to the two treatments whilst various values for p _MMF_ ranging between 0.55–0.75 in 0.05 increments were explored. The power for different sample sizes ranging from 40 to 250 using a one-sided type I error of 5% are provided in Figure 4.

Figure 4 shows that power increases for larger values of p _MMF_ as expected. Under the assumption that the efficacy of MMF is similar to MTX (both at 70% response rate), a sample size of 240 will yield adequate power for this study.

## Discussion

The overall objective of this study was to undertake a prior elicitation process with international experts to determine the efficacy and tolerability of MTX and MMF in JLS. It is evident from the resulting consensus distributions that considerable uncertainty remains concerning the potential merits and demerits of MTX and MMF. This Bayesian approach did reduce the sample size from 320 (predictions within a frequentist trial) to 240. However, for such a rare disease, this sample size may still not be feasible. We attempted to use a composite primary outcome encompassing both efficacy and tolerability, which was informed by patients and families. Unfortunately, the arising calculated sample size using efficacy alone as a primary outcome of 240 was high, and would be higher still if a composite score of both efficacy and tolerability was used. A composite primary measure does not therefore appear feasible. Involving patients and their families in the design of a future clinical trial with efficacy as the primary outcome, and adequately capturing tolerability as a key secondary outcome, offers the best opportunity for robustly evaluating further the benefits (or otherwise) of these two medications in the management of JLS. 

The uncertainty by experts around response of a medication is not unusual within prior elicitation exercises and was seen in the MYPAN study also ^33^. However, other data sources which provide evidence to reduce this uncertainty can then be combined with prior opinion to reduce uncertainty and allow modelling of a lower sample size. This approach was used in the MYPAN study. 

Since the prior elicitation exercise, two further retrospective studies provide further evidence for the efficacy, safety and tolerability of MMF in LS. A case series of MMF in 22 patients with JLS compared to 47 on MTX showed no significant difference in relapse-free survival between the groups although MMF appeared to induce more persistent remission, and MMF was well tolerated ^40^. In a study of 77 participants (all 16 years or older), MMF was well tolerated with 35% achieving disease remission ^52^. Both studies concluded that further controlled studies are needed. Statistical analyses of data from these studies may allow reduction of the sample size and a subsequent more feasible trial.

The international experts demonstrated through their prior distributions that they are more certain about the efficacy and tolerability of MTX compared to MMF, most likely relating to their familiarity with MTX as it has been considered ‘standard of care’ for JLS for some time. There was general consensus that whilst efficacy of MTX and MMF may be similar, MMF is superior in terms of tolerability. If these results could be further supported through clinical trial data, this could provide the first steps to changes in current treatment protocols for JLS.

There was unanimous support for the mLoSSI as the measure of efficacy in a clinical trial. However, within expert group discussions, there was some concern that a mLoSSI score of 0 (definition of efficacy) may be difficult to achieve. There was also consideration given to whether head and face involvement alone should be excluded because of perceived concerns regarding a lower mLoSSI score in this group of children at baseline. However, data presented from the National Registry of Childhood Onset Scleroderma (NRCOS) and Morphea in Adults and Children (MAC) cohort studies ^51^ showed that the median mLoSSI was 3 in children (interquartile range (IQR) 0–7) compared to 6 (IQR 0–17) in adults in the combined cohort (n=944; 496 JLS and 443 adult onset LS). The median mLoSSI at baseline in children with non-head lesions was 7 (IQR 5–12) in NRCOS (n=41) and 6.5 (4–12) in MAC (n=48), compared to head only lesions where median mLoSSI was 3 (2–4.5) in NRCOS (n=11) and 4 (3–5) in MAC (n=14). Although those with head only LS had a lower starting baseline, both head and non-head achieved a mLoSSI score of 0 during follow-up by 6 months across both cohorts. Consensus was achieved to include isolated linear head subtype and to use mLoSSI score of 0 as efficacy end-point at 12 months.

Exclusion criteria were not exhaustively defined but in group discussions it was agreed that this would include participants where the main indication for starting a DMARD was due to extra-cutaneous manifestations such as uveitis or arthritis as the primary efficacy outcome (mLoSSI) only measures skin involvement.

Considerations on CS regimes for a clinical trial setting included the difficulties with a mixed oral and intravenous regime within a clinical trial setting (oral route easier to administer in a multi-centre study) and the importance of limiting the duration of CS to ensure the efficacy of MTX and/or MMF is not masked by the responsive of JLS to CS. This led to consensus regarding regime B as preferred option which includes oral administration only for the shorter duration of 8 weeks.

Review of the literature preceding the consensus meeting showed a lack of a robust patient-reported outcome for measuring tolerability. The MTX intolerance severity score was reviewed but as this tool has been developed specifically for intolerance related to MTX, it was felt it would not identify some of the more common side-effects of MMF and would add bias to a composite primary endpoint ^6^. The Localised Scleroderma Quality of Life Instrument (LoSQI) has been designed in partnership with patients and does include a medication subscale ^53^. However, items in the scale were felt to under-represent MMF related side-effects and be weighted towards impact from CS and MTX. This highlights the lack of a generic drug tolerability tool for use in clinical trial settings, when tolerability is being considered as an important end-point.

## Conclusions

We have demonstrated international consensus for a clinical trial of MTX versus MMF in JLS with agreement on primary outcome measure, CS regimes and important inclusion and exclusion criteria. The prior elicitation process showed there was on-going uncertainty over the combined outcome of efficacy and tolerability of MMF versus MTX, highlighting the need for additional clinical data to facilitate future clinical trials.

## Data availability

Figshare. JLS Elicitation Extended data.pdf. DOI: https://doi.org/10.6084/m9.figshare.14994486.v1 ^54^

This project contains the following underlying data:

- JLS Elicitation Extended data.pdf (Extended data from methodology Appendix S1 Participants in prior elicitation meeting; Appendix S2 – Inclusion criteria for a clinical trial of MTX and MMF defined before elicitation exercise; Appendix S3 Detailed methodology of elicitation process.

Data are available under the terms of the Creative Commons Zero "No rights reserved" data waiver (CC0 1.0 Public domain dedication).

Data on R shiny application used in this study is available on request.
