# Supplementary material for: Prior elicitation of the efficacy and tolerability of Methotrexate and Mycophenolate Mofetil in Juvenile Localised Scleroderma
Source: AMRC Open Res. 2021 Sep 9;3:20. [Version 1] doi: 10.12688/amrcopenres.13008.1 (PMC11064983; doi:10.12688/amrcopenres.13008.1)
Supplement: Peer review history

# Reviewer report: Reviewer response for version 1

**DOI:** 10.21956/amrcopenres.14083.r26901

**Published:** 2022-03-07

**Authors:** Sandy Hong

**Affiliations:** 1 Department of Pediatrics, Division of Rheumatology, University of Iowa Stead Family Children's Hospital, Iowa City, IA, USA

Summary:  This article was a report of pre-trial expert consensus and modeling with a Bayesian approach to decrease the "N" or power (number of subjects) needed to perform a clinical trial to statistically evaluate the efficacy of MMF versus MTX for the treatment of juvenile Localised Scleroderma.

 It basically showed that even with a large group consensus that MMF works- the N for a trial is still large if using only efficacy as an endpoint. The authors suggest tolerability must be included as an endpoint.

 The article was very well written. It can be improved slightly in the conclusion section.

 My questions to help inform the conclusion or if the conclusion can allude to:

1. Where are they going to get more clinical data to facilitate a future clinical trial?  Will they use an existing registry? What about the development of a pilot Consensus Treatment Trials across their multiple international institutions with physician and patient entered data about efficacy and tolerability?

Is the work clearly and accurately presented and does it cite the current literature?

Yes

If applicable, is the statistical analysis and its interpretation appropriate?

Yes

Are all the source data underlying the results available to ensure full reproducibility?

Yes

Is the study design appropriate and is the work technically sound?

Yes

Are the conclusions drawn adequately supported by the results?

Yes

Are sufficient details of methods and analysis provided to allow replication by others?

Yes

Reviewer Expertise:

linear scleroderma

I confirm that I have read this submission and believe that I have an appropriate level of expertise to confirm that it is of an acceptable scientific standard.

# Reviewer report: Reviewer response for version 1

**DOI:** 10.21956/amrcopenres.14083.r26789

**Published:** 2021-09-24

**Authors:** Marija Jelusic, Mario Sestan

**Affiliations:** 1 Department of Pediatrics, School of Medicine, University of Zagreb, Zagreb, Croatia; 2 Department of Paediatrics, University of Zagreb School of Medicine, Zagreb, Zagreb, Croatia

The manuscript of Desai et al. represents the results of an international consensus meeting on the relative efficacy and tolerability of MTX compared to MMF in the treatment of juvenile localised scleroderma to develop the design of a potential future trial comparing MMF to MTX.

 The subject of manuscript is relevant since the treatment of juvenile localised scleroderma is challenging and there is a lack of randomised trials. 

 The abstract of the manuscript is clear and comprehensible, it contains general aims of the research and these are to undertake a prior elicitation process with international experts to determine the efficacy and tolerability of MTX and MMF in the treatment of juvenile localised scleroderma and to achieve consensus agreement on critical aspects of a future trial. The methods used in the research are also briefly presented, and at the end of the abstract the expected contribution of the research is described. All the data presented in the abstract are described in detail in other parts oof the manuscript. The abstract is written in such a way that it is clear to the general medical public what the manuscript is about.

 The introduction is coherently written and the literature references are relevant for the proposed subject of research. In the introduction the authors presented all relevant research and data needed to follow-up and understand other parts of the manuscript. The authors successfully summarized all pertinent knowledge to date related to the therapy of juvenile localised scleroderma with MTX and MMF.

 The general aims of the manuscript are clear and concise. The research is designed so that the general goals are achievable and accessible. The goals were achieved due to well-planned international consensus meeting.

 The section "Methods" understandably describes what has been done. The methods used in the manuscript are presented plainly. The authors used proven approach previously well described in the literature and used in paediatric rheumatology clinical trials such as the MYPAN trial. 

 The data were interpreted using appropriate statistical methods, which is briefly explained. Applying the above methods listed aims were achieved. The results are distinctly presented accompanied with three figures and three tables. Based on the presented results, it can be concluded that manuscript provides scientific contribution to problems stemming from the authors' work. These results may represent the starting point for future Bayesian approach clinical trial. 

 The discussion is concise and all important results are analysed in detail.

 We have a few questions for authors where the answers to them do not necessarily have to be included in the manuscript itself. 

 The introductory part of the manuscript provides data on incidence of juvenile localised scleroderma in the UK and a prevalence in the USA, followed by an illustrative example how with the number of new cases in the UK it would take at least 15 years to complete the study to allow direct comparison of the efficacy and tolerability of the two treatments. Since prevalence and incidence data differ in different parts of the world, can the authors provide data for some other parts of the world that would be of interest to readers?

 In the manuscript the authors presented their dilemma whether to exclude a group of children with head only localised scleroderma. Since there are several subtypes of the disease and it can affect different tissue depths, do the authors believe that the effectiveness of drugs may be different depending on the subtype, or depending on the extent of the disease and the depth of tissue involvement?

 Furthermore, do the authors consider that the duration of the disease before starting therapy may affect the effectiveness of drugs or the difference in effectiveness in the context of the fact that MMF may have an effect on both the inflammatory and fibrotic aspects of this disease? Would drug efficacy considerations change for patients with longer disease duration or with advanced disease?

 The authors stated that they took into account representation from UK and non-UK sites when forming the expert group, and in the Extended data that at least two representatives were dermatologists. From the Figure 2 it was concluded that the experts were more certain about their opinions on MTX rather than MMF. Were there significant differences of opinion as to whether the experts came from the UK or outside the UK or whether they were dermatologists or rheumatologists?

 Since one of the ultimate goals of future research is to suggest which drug should be the first choice of therapy, MTX or MMF, do the authors consider whether the variable of prices and availability of both drugs to the patients in different countries should be considered - whether the MMF justifies better or the same effect and fewer side effects compared to MTX to be recommended as first line therapy?

 Finally, we would like to thank the authors for their interesting manuscript and the opportunity to present our comments and wish them much success in future research on juvenile scleroderma, where therapeutic options are still quite limited and treatment alone is a major challenge in clinical practice.

Is the work clearly and accurately presented and does it cite the current literature?

Yes

If applicable, is the statistical analysis and its interpretation appropriate?

Yes

Are all the source data underlying the results available to ensure full reproducibility?

Yes

Is the study design appropriate and is the work technically sound?

Yes

Are the conclusions drawn adequately supported by the results?

Yes

Are sufficient details of methods and analysis provided to allow replication by others?

Yes

Reviewer Expertise:

Paediatric rheumatology, especially vasculitis.

We confirm that we have read this submission and believe that we have an appropriate level of expertise to confirm that it is of an acceptable scientific standard.
